# Gender and the Symptom Experience before an Atrial Fibrillation Diagnosis

**DOI:** 10.1177/0193945921999448

**Published:** 2021-03-12

**Authors:** Ryan E. Wilson, Kathy L. Rush, R. Colin Reid, Carol G. Laberge

**Affiliations:** 1 School of Nursing, The University of British Columbia, Okanagan Campus, Kelowna, BC, Canada; 2 School of Health and Exercise Sciences, University of British Columbia, Okanagan Campus, Kelowna, BC, Canada

**Keywords:** atrial fibrillation, gender, illness behavior, symptom, pre-diagnosis, treatment-seeking delay, symptom experience, symptom self-management

## Abstract

Atrial fibrillation (AF) is the most common arrhythmia in the world. Despite the increasing prevalence, there remains a limited understanding of how the pre-diagnosis symptom experience varies by gender. The purpose of this study was to retrospectively explore gender differences/similarities in the pre-diagnosis period of AF. Twenty-six adults (13 men and 13 women) were interviewed guided by the Symptom Experience in AF (SEAF). Data were analyzed using a two-step approach to thematic analysis. Women had greater challenges receiving a timely diagnosis, with 10 women (77%) experiencing symptoms ≥1 year prior to their diagnosis, in comparison to only three (23%) of the men. Women also reported more severe symptoms, less AF-related knowledge, viewed themselves as low risk for cardiovascular disease, and described how their comorbid conditions confused AF symptom evaluation. This study provides a foundational understanding of differences/similarities in the AF symptom experience by gender.

Atrial fibrillation (AF) is the most common arrhythmia in the world, affecting men and women in unique ways (Walfridsson et al., 2019). AF prevalence has increased by 13% over the past two decades, with an estimated 33.5 million people worldwide living with the condition and five million new cases diagnosed every year (Chugh et al., 2014; Lip et al., 2016) The incidence of AF among men is one-and-a-half times the rate among women; however, given the greater longevity of women, the absolute number of men and women with AF is similar and more common in women 75 years and older (Ko et al., 2016; Michelena et al., 2010; Norris et al., 2020). AF predisposes both genders to serious risks, including stroke, dementia, and heart failure (Camm et al., 2012; January et al., 2014; Kirchhof et al., 2016). However, female sex is an independent risk factor for AF-related stroke, and strokes are more common (for those aged 75 years and older) and more severe (greater neurological deficits after a stroke) among women with AF compared to men with AF (Kassim et al., 2017; Norris et al., 2020).

There are pronounced sex and gender differences in men’s and women’s experiences of AF symptoms (Walfridsson et al., 2019). AF symptom prevalence, persistence, and burden are greater for women than for men (Blum et al., 2017). Females have higher resting heart rates, corresponding to more rapid, uncontrolled rates of AF and more severe symptoms (Ko et al., 2016; Michelena et al., 2010). Female sex has been shown to be an independent predictor of palpitations, dizziness, dyspnea, fatigue, and chest pain (Blum et al., 2017). More recent evidence indicates that there is a greater clustering of symptoms in females (Streur et al., 2017). The influence of gender may account for the differences in the types of symptoms women choose to report, in their symptom self-management, and health care–seeking responses (Bartz et al., 2020; Reynolds et al., 2006). Even though women at a biological sex level experience more severe and frequent AF symptoms, the lengthened treatment-seeking time frames among women are likely the interaction of gender differences in symptom perception and evaluation (i.e., how symptoms are communicated, for what reason, and to whom) (Bartz et al., 2020; Davis et al., 2013).

Well-known gender disparities exist in the treatment and the care that men and women who are newly diagnosed with AF receive. Women have greater difficulty in obtaining an AF diagnosis, at times, because of atypical symptom presentation, delaying diagnosis and care (Ko et al., 2016), resulting in women being under treated in terms of both rate and rhythm control (Potpara et al., 2012), and are less likely to receive catheter ablation used to treat irregular heart rhythms (Bhave et al., 2015; Walfridsson et al., 2019). Although at greater risk for AF-associated strokes, women less often receive anti-coagulation therapy, and experience increased door-to-imaging times (the time perod between arrival to the emergency department and the commencement of cerebral imaging) when presenting with stroke symptoms, leading to greater numbers of ineligibility to receive thrombolytics (Bartz et al., 2020; Madsen et al., 2018). As a result of these gender differences, women with AF suffer from more symptoms on a daily basis (having been undertreated) and experience a decreased quality of life (QOL) compared to men (Reynolds et al., 2006; Volgman et al., 2009; Walfridsson et al., 2019).

Compared to the number of research studies exploring sex differences in AF, there is a paucity of data regarding gender differences in the pre-diagnosis symptom experience or how individuals perceive, evaluate, and respond to their symptoms. The scarcity of evidence regarding gender and AF symptom experiences continues to be a problem despite evidence of the important role gender plays in the evaluation and response to symptoms in patients experiencing other cardiovascular disease (i.e., myocardial infarction, heart failure, and stroke) (Davis et al., 2013; Lichtman et al., 2015; Noureddine et al., 2010). Only two studies within the AF population (Deaton et al., 2003; McCabe et al., 2016) have explored the symptom experience in AF. Of these two studies, only Deaton et al. report differences in symptom experience by gender, finding that women’s health care providers (HCPs) often did not take their AF symptoms seriously, either dismissing them or attributing them to menopause or stress. Outside of cardiovascular research, the evidence is mixed regarding the influence of gender on the symptom experience (Fouladbakhsh & Stommel, 2010; MacLean et al., 2017). Given the absence of data within the AF population, more research is needed to understand the gender differences in the symptom experience of AF. Understanding gender differences/similarities during this critical pre-diagnosis period can minimize the effects of a delayed AF diagnosis on QOL, morbidity, and mortality.

## Purpose

The purpose of this study was to retrospectively explore gender differences/similarities in the pre-diagnosis period of AF, which is defined as the time from patient’s recognition of the onset of symptom(s) to the time of AF diagnosis by a health care provide. This study was broadly guided by the Symptom Experience of patients with Atrial Fibrillation (SEAF) model (Wilson et al., 2020), which is a hybrid model of the symptom management model (Dodd et al., 2001) the symptom experience model (Bruno, 2013), and the results from the original analysis with this dataset. The SEAF posits an intricate process of experiencing symptoms through a process perceiving or noticing symptoms, evaluation or speculating on possible causes to form a self-derived theory, and forming a response. Overarching influences on the entire SEAF include gender, age, knowledge, expectations, and symptom characteristics. Such models have not been previously considered in understanding the AF pre-diagnosis period. This article reports on the gender differences observed within the original study by Wilson et al. (2020), exploring the entire SEAF.

## Methods

### Design

This study used interpretive description (ID), a qualitative method developed by Thorne et al. (2004), as a way to generate clinically relevant knowledge for health disciplines.

### Sampling

Recruitment commenced following a joint ethics review from a university Behavioral Research Ethics Board and a Regional Health Research Ethics Board (H15-02443). Eligible patients with a diagnosis of AF in the last ≤12 months (before study commencement), who were ≥19 years of age, were recruited through convenience sampling. A sample size ranging from 5 to 30 participants is considered appropriate in ID, if the phenomenon under study occurs commonly (in this case, AF symptoms; Thorne, 2016). Recruitment at the upper end of this sample size requirement/prescription (*n* = 26) was necessary in our study to allow for comparison between men and women (Thorne et al., 2004). Participant recruitment occurred in two primary locations—an AF clinic and Rapid Access to Cardiac Evaluation Clinic. An HCP at each site provided information about the study and a permission to contact letter to interested and eligible patients. Patients who signed the permission to contact forms received a phone call from a research team member (RW) to provide additional information about the study, answer questions, and arrange a time for an interview. Exclusion criteria included cognitive impairment (e.g., dementia), uncompensated hearing impairment, post-op AF, athletic AF, and/or patients who lived further than 90 kilometers from the university. Cognitive impairment was screened for using the Mini-Cog, with scores of ≤2 excluded from the study (0, 1, or 2 impaired; 3, 4, or 5 impairment less likely or absent) (dementia sensitivity 76%–99%; dementia specificity 89%–93% with a 95% confidence interval) (Borson et al., 2000). Patients provided signed consent prior to the start of the interview.

### Data Collection

Semi-structured interviews were the primary method of data collection. These in-person interviews were carried out by an experienced interviewer with a background in cardiology. The digitally recorded interviews lasted 60–90 minutes and occurred in various locations, including participants’ homes, offices, or coffee shops. Men and women were both asked questions designed to explore the pre-diagnosis symptom experiences, focused on their perceptions, evaluations, and responses to symptoms (see Table 1). Follow-up and probing questions allowed for clarification and elaboration of experiential details. As per ID, multiple data collection tools were administered as a way to further understand the phenomena and triangulate the findings (Thorne, 2016). These two other data collection tools were administered prior to the interview questions and included the Symptom Checklist (SCL) and a locally developed demographic and health history questionnaire (Bubien et al., 1993). The SCL is a list of 16 common symptoms associated with AF (e.g., palpitations, dyspnea, and dizziness) and rated according to frequency (0 = never, 4 = always) and severity (1 = mild, 4 = extreme) (Cronbach’s α = 0.87–0.92 for the symptom frequency scale and 0.89–0.93 for the symptom severity scale) (Bubien et al., 1993; Jenkins, 1993; Jenkins et al., 2005). The SCL provided the interviewer with an overview of the type, severity, and frequency of symptoms experienced, which assisted the researcher to further probe the symptom experiences. The demographics and health history questionnaire included the collection of data that was then used to compute the CHA_2_DS_2_-VASc (Andrade et al., 2018), a tool which predicts the overall risk of stroke based on risk factors, which include heart failure, hypertension, age, diabetes, prior stroke or transient ischemic attack (TIA), vascular disease, and sex.

**Table 1.** Semi-Structured Interview Guide.

Perception	Evaluation	Response
• Can you tell me your story, how you ended up being diagnosed with AF? Let’s start from the beginning? When did you begin to experience changes in your body that something was not as it should be? What symptoms did you experience? • Looking back, what did you first notice about your symptoms or illness? When was this? How aware were you of the bodily or psychological changes that were occurring from your AF? • How frequently did the symptoms occur? Were there some symptoms more intense than others? Did any of the symptoms cause any distress?	• Once you were aware of the symptoms, how did you decide what they were resulting from, or what they were an indication of? • Where did you turn to for knowledge or information? • Who, if anyone, did you talk to about how you were feeling? • What did you think was the cause of this?	• How worried were you about your situation? What did you think would come of it? • Can you tell me how you first responded to the symptoms? Did you change anything you were doing (activities) in your daily life? Did you try to alter anything else in your life (e.g., diet)? • Is there anything in your childhood or upbringing or culture that might have influenced how you respond in illness? • Did you experience any emotions as a result of your symptoms? • Tell me about what made you decide to seek treatment from the medical system? • What do you recall as being an important factor(s) in deciding about your treatment? • When making decisions about treatment, how did this experience differ from other experiences that you may have had before? • Tell me about what made you decide to seek treatment from the medical system? • How did your perceived consequences of the AF effect your decisions on treatment?

*Note*:.*AF = Atrial fibrillation.

### Analysis

A medical transcriptionist transcribed all interviews verbatim; each interview transcript was compared to the audio recording to ensure accuracy of the interview data by the lead researcher. All data were uploaded to NVivo (11.4.1) for coding, and pseudonyms were used instead of patient names. Using a two-step deductive/inductive approach to thematic analysis (Fereday & Muir-Cochrane, 2006), transcripts were, first, coded deductively, identifying units of text representing each of the central concepts within the SEAF (perception, evaluation, and response) (Wilson et al., 2020). In the second step, inductive coding expanded upon the meaningful units of text identified in step one, aimed at understanding the broader experiences within each of the central concepts of SEAF. Full details of the analysis can be viewed in a previous paper by Wilson et al. (2020). All codes developed in the two-step approach to coding were condensed and synthesized into themes and sub-themes, which reflected differences and similarities by gender. Multiple coders (RW, KLR, and LH) coded the first three transcripts to ensure consistency across the codes, and a coding schema was created and applied to the remaining interviews coded by the lead researcher. Ongoing discussions continued with all team members as the analysis unfolded. Analysis was undertaken concurrently with data collection and continued until recurring themes emerged. Once all interviews were coded, we compared and contrasted all codes by completing a matrix analysis with which we compared the frequency of codes and the relationships between/among men and women (Averill, 2002; Lindsay, 2019; Whitley, 2016). Demographic data were summarized using SPSS 24. To test for differences between men and women in the data obtained from the SCL, an independent sample *t*-test was completed using SPSS 24.

The rigor of this qualitative study was addressed through several different strategies, including prolonged engagement using thorough in-depth interviews with the option of follow-up, extensive examples of each theme (persistent observation), triangulation of data through multiple data collection strategies (SCL, interviews, field notes), and multiple coders (Korstjens & Moser, 2018; Thorne et al., 2004). Reflexivity in the research process was facilitated through journaling, which examined the lead researchers explicit and implicit assumptions, preconceptions and values, and how these affected research decisions (Korstjens & Moser, 2018; Thorne, 2016). Lastly, transferability of the research was established by providing sufficient data or evidence in the findings to allow for an informed judgment as to whether the findings are applicable in another context or population.

## Results

The study sample consisted of 26 participants, all of whom had received an AF diagnosis within the 12 months prior to the interviews. Participants self-identified as men (*n* = 13) or women (*n* = 13), all Caucasian, ranging in age from 43 years to 85 years; the mean age for men was 68 years and, for women, it was 66.3 years (see Table 2). Both men and women had similar mean CHA2DS2-VASc Scores of 2.23 and 2.77, respectively. Women had greater challenges than men in receiving a timely diagnosis. Within perception, women reported more severe symptoms, less AF-related knowledge, viewed themselves as being at low risk for cardiovascular disease, and reported more confounding symptoms (e.g., fatigue, difficulty sleeping) associated with other comorbid conditions. Women also experienced the longest pre-diagnosis time frames, with 10 women experiencing symptoms for a period of time greater than or equal to one year, in comparison to only three men. Only two women received a diagnosis within 48 hours of first noticing symptoms compared to four men. Each of these sex and gender differences in the symptom experience are compared and contrasted throughout the following section. The results are organized according to the overriding themes of perception, evaluation, and response.

**Table 2.** Demographic and AF Participant Characteristics.

Characteristic	Men (*n* = 13)		Women (*n* = 13)	
	*N*	%	*n*	%
Marital status				
Single	1	8	0	0
Married	11	85	8	62
Widowed	0	0	2	15
Separated/divorced	1	8	3	23
Current work position				
Retired	11	85	7	54
Clerk	0	0	1	8
Health care	1	8	4	30
Service	1	8	1	8
Comorbidities				
Arthritis	4	31	5	38
CHF	1	8	1	8
Diabetes	2	15	0	0
Dementia	0	0	0	0
Hypertension	4	31	5	38
Sleep apnea	2	15	2	15
Hypothyroid	2	15	2	15
COPD	2	15	1	8
Stroke/ TIA	3	23	1	8
Depression	1	8	0	0
Anxiety	1	8	1	8
MI	0	0	1	8
Vascular disease	0	0	1	8
Other	3	23	4	31
No comorbidities	2	15	3	23
Living arrangements				
Live alone	0	0	1	8
Live with children (no partner)	0	0	2	15
Live with partner	11	85	9	69
Live with other (family, friend, etc.)	2	15	1	8
Race				
Caucasian	13	100	13	100
Knowledge of AF prior to diagnosis				
Described as good knowledge of AF	0	0	2	15
Heard of it but little understanding	9	69	7	54
Never heard of it	4	30	3	23
Education				
Partial high school	3	23	0	0
Completed high school	3	23	3	23
Some college	3	23	6	46
College‎/university graduate	4	30	4	30

*Note*. AF: Atrial fibrillation; CHF: congestive heart failure; COPD: chronic obstructive pulmonary disease; TIA: transient ischemic attack; and MI: myocardial infarction.

### Perception: Severity, Frequency, and Types of Symptoms

Participant perceptions recounted the process of their noticing or becoming aware of previously unfamiliar body sensations and symptoms. Comparatively, both men and women were similarly aware or unaware of their pre-diagnosis symptoms. However, perceptions of the severity, timing, types of symptoms, and the language they used to describe symptoms differed between men and women.

Women, more so than men, described intense, burdensome symptoms, which, at times, incapacitated their abilities to perform activities. For example, some women experienced such intense symptoms that they had to stop the activities they were engaged in (e.g., activities of daily living [ADL], housework, walking, sports). Gillian’s story highlights the experiences of many of the women who reported extreme symptoms during their pre-diagnosis period:

But I went in, went to the bathroom. And then when I stood up again I just felt so dizzy. So, I sat down on the floor. And then next thing I woke up lying flat on my back. A bump on my head from hitting my head.

Using an independent samples *t*-test, a significant difference in the severity with which AF symptoms were reported on SCL was identified between men and women (see paper by Wilson et al. [2020] for full results of SCL). These results corroborated the intensity with which women experienced AF symptoms, reporting more severe (M = 19.5 vs. M = 13.6; *p* = 0.02; maximum score 48) and frequent (M *=* 20.3 vs. M *=* 16.7; *p* = 0.14; maximum score 64) symptoms than men. No statistically significant differences were detected among individual symptoms experienced.

Women not only experienced a wider range of symptoms than did men but some of their symptoms differed from men. As regards pre-AF diagnosis, only women reported perceiving gastrointestinal (GI) symptoms, such as nausea, vomiting and diarrhea, and abdominal pain. These symptoms often co-occurred with severe dizziness or syncope. Brenda reflected on the severe symptoms that led to her AF diagnosis:

The next symptom I felt was that I had to evacuate my bowel, which emptied out completely. . . I went and lay on my bed at which point I was still feeling very light-headed, very dizzy and I threw up my lunch.

Women also reported more frequent and severe syncope, or near syncope, than did men. Joyce collapsed while standing in line waiting her turn to vote, and both Lydia and Margret reported becoming faint and nearly blacking out during the normal ADLs. Of the men who were interviewed, only Clinton reported that he fainted while sitting in his living room chair.

More women reported fatigue, experienced difficulties falling and/or staying asleep because of their racing heartbeat and pulsing heart in their ears, and differed in the context with which their symptoms affected their activity. Donna described her difficulty, “Yeah. And even now when I lay on my left side there were a couple of nights I couldn’t sleep because my heart was going.” Lack of sleep left many participants experiencing daily fatigue. Lastly, men and women had different contexts with which they reported their activity intolerance. Women reported activity intolerance in relation to ADLs, such as walking, climbing stairs, doing yard work, or cleaning the house. In contrast, men’s context of activity intolerance was more often reported during sporting activities, such as golf or tennis. “I finally had to quit my tennis game because between serves I could not catch my breath” (Len).

Differences in their AF symptomatology were reflected in men’s and women’s pre-diagnosis experiential descriptions. Women used stronger and more descriptive language such as “pounding” or “heavy” to describe the feeling of their heartbeat. “No, it was a pounding, you know, pounding. They call it flutter. It’s more than a flutter. I could see my chest going up and down” (Anne). Helen likened her experience to that of having a racehorse inside her chest that was pounding back and forth. Finally, both Farah and Brenda used the term “pounding” when describing their heartbeat. Conversely, men used words such as “flutter” or “palpitations” to describe their symptoms: “Yeah. Just I could I’d say times I’d have a little bit of a flutter” (Kevin).

### Evaluation

Evaluation described participants’ cognitive and self-derived theorizing processes to make sense and explain their bodily sensations and symptoms. The following section explores how men and women not only differed in their self-derived theories but also in their overall level of concern, risk perception, and the urgency in their evaluation. This section also explores two influencing factors (reliance on others, knowledge of AF), which differed by gender.

#### Self-derived theories

Both men and women reported difficulty in making sense of and explaining their symptoms. However, it was mostly the women in the study who reported confusing, subtle, or elusive symptoms, which challenged their symptom evaluation. Margret’s reported experience exemplifies the challenges of searching for answers regarding the cause of her symptoms: “They just happen [palpitations], yeah. No rhyme or reason to it. There’s no pattern that I could pinpoint” (Margret). Ellen also queried her symptoms:

It was just bizarre. Just like I said we had been walking all day. Just, I mean busy all day doing stuff. I was putting dinner on the table and I just thought dang, I don’t feel very good. What am I getting sick? This is weird. And I thought it would just go away.

Women more than men explained their symptoms as stress-related due to the burden and stress of caregiving, their comorbidities, or from their own neglect of their health. Men had similar theories; however, compared with women, their stress-related theories were more situational and work-related, and they felt that their symptoms were more heart-related and acute in nature. Despite their uncertainty about the cause and meaning of their symptoms, all participants eventually formed tentative self-derived theories to explain their symptoms (i.e., physical deconditioning, diet, and chronic health conditions). Gender differences in self-derived theories are described in Table 3.

**Table 3.** Differences in Self-derived Symptom Theories.

Self-derived Theories	Description	Example
Stress-related theories (six women vs. three men)	• Regarded “stress or anxiety” as a transient cause of their symptoms that they anticipated would eventually disappear • Women talked about the burden and stress of caregiving as an explanation for their symptoms • Men’s stressors were more situational and work-related	“Our dog was sick. So, then I was dealing with getting the dog better. So, that day I went into atrial fib that was the day we had to put the dog down. And then the next Monday my husband went into the hospital with heart failure. And then he got a bed sore when he was there. He’s paraplegic. So, then he was home for a week. And then he had to go back into the hospital. And then that night I had another bout of it. So, that’s why I’m thinking it’s kind of stress related. And then my dad is out of town. So, I’m like trying to feel like I should go down there and help him out. And then my husband’s sick in the hospital. I’m not feeling very well because my heart’s not working well. And the dog just died. It’s like oh my God. So, yeah” (Gillian) “And the stress of moving to a new city, the stress of a new job, the stress of giving up the job and security and stuff. So, you know, it all made sense that, you know, this could be stress related” (Len)
It is my comorbid conditions (six women vs. two men)	• More women theorized that their AF symptoms were a manifestation of preexisting medical conditions, such as asthma, COPD, or MI • Men’s theories were more heart related and acute in nature	“I’d had two concussions in the previous couple of years. And I just thought it was, you know, something to do with the fact I’d had a couple concussions. I never thought it was something to do with my heart” (Cathy). “I always thought I was having an asthma attack but if I look back at it I was having more than that. I think it’s all related to this [AF]” (Donna) Men’s theories were heart related and acute in nature such as “heart attack or stroke” (Isaac), “palpitations” (Dan), or a “heart issue related to blood flow” (Edward)
It is my fault: self-blame (six women vs, five men)	• The language used by women to describe their weight gain was often very critical, harsh, and unforgiving • Men’s language was more matter of fact	“Yeah. Like just shut up and power through it [symptoms of AF?]. And then looking back like there was a couple times going up the stairs at home I would think God, you have gotten so fat. Now, you can’t even go up the stairs. Because I’d be really out of breath. And I was like, well, you’ve got to get rid of the weight. I kept, I blamed a lot on my weight” (Ellen) “I was perfectly convinced it was entirely because of the fact that I was actually overweight and having trouble getting up these hills which was making me breathless and therefore, you know” (Monty)

*Note*. *HF: Heart failure; COPD: chronic obstructive pulmonary disease; and MI: myocardial infarction.

#### Seriousness of symptoms

More women evaluated their symptoms of “little concern” or “nothing serious,” and of low priority or risk to their health. “I just thought that my heart is being stupid and so what. But then it would, you know it would just go away. I mean, it’s not like something bad” (Ellen). Cathy, who experienced severe dizziness, palpitations, and GI symptoms, described her lack of concern: “Just wanted to go home and crawl to bed. I actually didn’t care. You live, you die, oh well. Wasn’t too worried about it.” Women spent a considerable amount of time theorizing that their symptoms were not “worrisome” or “serious,” and, therefore, they could continue with normal life.

#### Perception of vulnerability

Six women in the current study described feeling immune to cardiovascular illness, while men unanimously saw themselves as having at least some cardiovascular risk factors. Women, more often than men, considered themselves very healthy, worked hard to maintain their health, and did not consider the possibility of cardiac disease in their symptom evaluation. Joyce and Lydia’s comments epitomize the views of many of the women in the study: “Yeah. So, no, it was actually the last thing on my mind because I always kept a healthy heart. Ate really healthy” (Joyce), and Lydia, “We didn’t know what it was because my entire life, I’ve been working out, you know, always, always.” Likewise, Margret held minimal expectations of developing heart disease because of her healthy lifestyle:

I probably would have put it down to no, not me. Because I never smoked, I don’t drink. I’m very healthy. I don’t eat a lot of junk food. Don’t do the deep-fried stuff. Every morning I either do stretching, or I do a small forty-five to an hour-long workout. I eat extremely healthy. I know that.

Women’s lowered expectations of cardiovascular disease were influenced by media portrayals of cardiovascular disease as being a man’s disease. Ellen minimized the significance of her symptoms as “no big deal,” in part, from the powerful messages she had seen in drug commercials:

I just thought it’s not that big of a deal. Like I’ve never heard ever of like I said before the Eliquis [AF medication] thing. And they’re guys. They’re not women. On all of the commercials on TV, it’s all men as a matter of fact. I don’t think there’s one woman on any commercial I’ve ever seen.

Although more women than men (6 vs. 0) indicated that they had no expectations of heart disease, two men who considered themselves to be healthy and fit were also unsure about the meaning of their symptoms. Ben, who self-evaluated as healthy was shocked to learn of his AF:

Yeah, yeah. Came as a total shock because I considered myself a moderate drinker, never smoked, never did drugs. You know, I eat well, do smoothies every morning. I thought I was pretty fit and pretty healthy.

#### Influencing factors on the evaluation process

There were two main factors that influenced men’s and women’s evaluation processes, partner reliance, and knowledge.

##### Role of others

Men and women demonstrated differences in the “when” and “why” they choose to disclose their symptoms to their spouse. Men talked about their new AF symptoms to their spouses, who often evaluated their seriousness and urged them to seek help. Monty’s wife urged him to see his physician to further evaluate his symptoms:

I talked to my wife about it [his symptoms]. She was the one that said you have to go to the doctor. You have to tell him about all the things you’ve got because he will actually look at you and see because there has to be something wrong.

Kevin described his wife as “more skittish or concerned” about his symptoms than he was, indicating he followed her suggestion to seek care, stating: “You should never argue with a nurse. Especially if the nurse is your wife.” Of the few women who consulted their spouses, they were met with a lack of partner concern from their male partners who minimized their symptoms. Helen finally told her spouse about her symptoms when she could no longer keep up with him during their daily walks because of shortness of breath (SOB), only to have him reinforce her denial, “Well, I guess he was like me because it was his mom who had the same kind of thing [fatigue and SOB]. And he figured it was just nothing wrong. So, you just get up and deal with it.”

##### Knowledge differences

Men and women differed in their overall reported knowledge about AF (i.e., typical signs, symptoms, and treatments), with nearly three times as many women stating that they were unfamiliar with AF before their diagnosis. Despite higher levels of educational attainment, women’s lack of AF knowledge impaired their ability to accurately evaluate their symptoms. Ellen, who experienced symptoms for over nine months before her diagnosis, recalls how her doctor was shocked that she was unaware of her AF, “So, when the cardiologist came in he said, well, you have this thing called Afib, and he was shocked. He said, so you don’t, you didn’t know you had Afib. Like no. What is that?” Likewise, Anne who experienced many years of AF symptoms (palpitations), which were unaccounted for, described how even with her work experience in healthcare, she did not know what AF was: “Even though I’d worked on the cardiac ward too for a few months. I didn’t clue in, you know. No, I had no idea what those terms meant really, you know.”

### Response

Response involved all actions/activities that participants initiated in responding to their symptom(s). Men and women differed in their response to AF symptoms. The following themes explore some of the reasons and context surrounding the varied gender responses.

#### Self-managing symptoms

There were gender differences in how men and women self-managed their symptoms. Women demonstrated a greater propensity (8 vs. 2) to initiate self-treatment in response to their AF symptoms. The prolonged period of self-treatment extended the pre-diagnosis time frame, particularly when these measures were effective at suppressing their symptoms. One self-management strategy that women reported using more frequently was lying down and resting, following the onset of symptoms. The *need* to “lay down” also demonstrates the intensity and severity (e.g., dizziness) of their AF symptoms, as these women needed to lie down due to the severity of their dizziness (presyncope). Cathy’s experiences demonstrate how laying down on the ski hill was needed to avoid fainting: “Like almost fainting. That bad like when I was skiing, I just said to my girlfriend, oh, something’s wrong, you know. I said I just have to fall down.”

Over half of the participants self-managed their symptoms by modifying their physical activities—increasing, decreasing, or stopping activities all together. Although more women tended to restrict their activities, men modified their activities rather than avoiding them altogether. For example, Monty described how he had to cut down and adapt his golfing: “So, to start with I found it was getting more and more difficult to walk around the golf course, for instance. And so, I started taking carts.”

Finally, men, more than women, responded by deep breathing or coughing as a means to correct their palpating heart. John explains how this technique worked for him:

I’ll get a little attack once every two, three weeks but I can control it with breathing. I take big breaths in through my nostrils and breathe out slowly through my mouth. And I do that anywhere from five to ten times. And it’s gone.

Similarly, Clinton used coughing to correct his symptoms while on the golf course: “you just go (cough, cough) and take some deep breaths in between. And your heart will regulate and that’s what I would do. I did that for two or three years.”

#### Avoiding talking themselves out of being sick

Although both men and women described “not having time” to be sick, their reasons varied. Overall, more men than women talked themselves out of being sick because of their need to continue with active lifestyles:

But I said I still swim every day. I walk or run every day, do yoga at night for, you know, half an hour. We do yoga classes usually twice a week. We’ve been pretty busy now. And I think we just caught up with things. I didn’t have time to get sick. And you’re sort of in denial, you know. (Greg).

On the other hand, women described talking themselves out of being sick more often because of their many caregiving, family, and work obligations:

Because then you just don’t have time. Like, I don’t have time for this. Taking care of his parents and doing stuff for my husband. I’m doing the job. What the hell? I don’t have time. Having a cold, no, don’t have time for that. Don’t get sick. Don’t get the flu. There’s so much stuff to do all the time. Yeah, I just don’t have time for it. And you just talk yourself out of things, too, I think because you don’t have the time. Like well, I can’t be sick. (Ellen)

#### Challenges with health care professionals

In this study, 7 out of the 13 women indicated that repeated health care–seeking efforts were needed in order to obtain an official diagnosis. This experience was perpetuated when a diagnostic test returned normal, leading to several of the women feeling like their HCPs dismissed their symptoms. For the women, this added challenge in obtaining a diagnosis following health care seeking created frustration, anxiety, confusion, and anger. Farah’s experiences demonstrate how receiving normal diagnostic testing was still worrisome:

And then I didn’t know if there was something wrong with my heart or not so that was when I went to see my doctor. Yeah, I had the stress test and the heart ultrasound, and everything was normal. I was kind of worried because I thought even though they said it was normal I was thinking then why is it doing that. (Farah)

Not receiving effective health care when they sought it created tensions with their GPs as Anne described:

So, now I’m really angry at my doctor. I really accused her of my Atrial Fibrillation. I said, “This is all your fault. You’ve ignored it and you knew. And I told you several times.” And she said to me, “But you haven’t told me recently, have you?” And I thought what’s the point if you’re calling me paranoid.

Conversely, 3 out of the 13 men who reported repeat health care seeking were not as bothered by this experience. When questioned whether going to the emergency room bothered John, he responded by saying “No, not really. I actually felt a little relieved they couldn’t find anything, because I thought okay maybe it’s not my heart. I’m thankful for that.”

## Discussion

Men’s and women’s pre-diagnosis symptom experiences were marked with several differences in how they perceived, evaluated, and responded to their symptoms. In our study, it was women who endured the longest pre-diagnosis time frame, and yet also perceived more frequent, severe, and wide-ranging symptoms. Women also reported lower levels of worry, a greater propensity to engage in self-treatment strategies, derived more non-illness interpretations of their symptoms, and tended to underestimate the severity of their symptom(s). Recent cardiac literature confirms that there are pronounced differences in men’s and women’s experiences of AF symptoms (Walfridsson et al., 2019), with more women reporting palpitations, dizziness, dyspnea, fatigue, and chest pain associated with AF (Blum et al., 2017). However, no other studies have identified the same complexities of symptom evaluation that many of the women in our study uniquely faced during the pre-diagnosis period.

A recent study exploring the accuracy of patients’ perceptions of their prevailing heart rhythm concluded that women underestimate their AF symptoms as compared to men (Garimella et al., 2015). This minimization of symptoms may be linked to gendered cultural norms, whereby women attend to the needs of others at the expense of their own health (Bartz et al., 2020; Clarke & Bennett, 2013; Lichtman et al., 2015). Furthermore, failure to timely access health care for their symptoms maybe the result of competing demands or conflicting priorities, which influenced their perception of their symptoms (Lichtman et al., 2015). This tendency to minimize or underestimate cardiovascular symptoms is consistent with current findings in which women justified delayed help-seeking because of caregiving priorities.

Compared to men, symptom evaluation was much more challenging and complex for women who participated in the current study. Women expressed being less vulnerable (or at less risk) to cardiovascular disease, interpreted their symptoms to be associated with non-illness reasons (e.g., too much stress), or the overlap of symptoms associated with other comorbid conditions. Evidence has shown that women with cardiovascular disease symptoms often experience atypical symptoms (i.e., fatigue, epigastric discomfort) that are attributed to other comorbid conditions that can confound symptom evaluation (Bartz et al., 2020; Gallagher et al., 2010; Norris et al., 2020). Women’s lowered expectations of developing cardiovascular disease are thought to be deeply rooted in the gendering of heart disease as primarily a man’s disease (Emslie & Hunt, 2009). Furthermore, women in the current study were more deficient in AF knowledge, which may account for their increased challenges in evaluation and assessment of their symptoms (Frewen et al., 2013; Goli et al., 2012).

There are clear gender differences in when and how men and women disclose their symptoms to their partners, following the first awareness. Men’s greater disclosure of their symptoms with their female partners and the support they received for symptom evaluation and response are evident in other cardiac literature (Noureddine et al., 2010; O’Brien et al., 2005). Partners’ insistence, that men be evaluated by an HCP, was the impetus for early treatment seeking among many of the male participants. One explanation is men’s smaller support networks to aid in their evaluation of symptoms, whereas women routinely consult friends rather than their partners in the face of illness (Schoenberg et al., 2003).

Looking to gender differences in other literature provides some possible answers as to why many women in the current study continued with self-treatment activities, enduring a longer pre-diagnostic time, despite severe symptoms, such as dizziness and syncope. Women are more likely to suffer financial barriers limiting access to care, describing problems arranging effective transportation and caring for others over self as impediments to care (Bartz et al., 2020). A meta-analysis exploring the symptom experiences of women with chronic illness found that women responded to frightening symptoms, such as chest pain, with more avoidance-type coping strategies (O’Neill & Morrow, 2001). Other studies conducted with cardiac patients suggest that women’s tendency to initiate self-treatment activities stems from wanting to maintain control and from a perception of the risks associated with cardiovascular disease as being low—a finding supported in our study (Lichtman et al., 2015). No other study has reported on gender differences in the self-management of AF symptoms that occur and are self-detected prior to medical diagnosis. Consequently, our study is the first to report that women with AF symptoms engage in self-management to a greater degree than men do, even while experiencing more severe symptoms.

Many women felt their symptoms were dismissed when they sought health care. Although not gender-specific, the dismissal of AF symptoms has been observed in other recent AF literature (McCabe et al., 2015, 2016; Withers et al., 2015). Gender inequality has also been previously documented in relation to post-diagnosis AF care, with women reporting lower QOL and more severe symptoms, while being undertreated in terms of rate control, anticoagulation, electro-cardioversion, and catheter ablation (Bhave et al., 2015; Blum et al., 2017; Piccini et al., 2016; Potpara et al., 2012; Schnabel et al., 2017; Walfridsson et al., 2019). It is important to acknowledge the clinical importance of early treatment seeking, equal access to diagnostics for both men and women, and the implementation of anticoagulants for eligible patients post AF diagnosis. Many of the risks associated with AF, such as thromboembolic strokes, heart disease including heart failure and myocardial infarctions, dementia and even death, can be minimized if patients seek and receive treatment soon after the onset of symptoms (Camm et al., 2012; Frewen et al., 2013; January et al., 2014; Kirchhof et al., 2016).

Although results of this study provide a novel, in-depth look at the experiences of men and women prior to an AF diagnosis, it does have several limitations, including sample composition, which lacks ethnic and cultural diversity (all White sample; limited rural representation: *n* = 1). Additionally, the transferability to other populations may be limited by the choice of recruitment sites, with all participants having been recruited through hospital-based programs. Lastly, interviews may be subject to recall bias, as participants were recalling events occurring weeks to several years previous to their participation in the study.

This study provides a foundational understanding of differences/similarities in the AF symptom experience by sex and gender. Further research is needed to cultivate gender-sensitive ways to support men and women to seek timely care, to identify the barriers to health care seeking and timely diagnosis, and to develop gender-sensitive symptom assessments, which recognize that men and women experience, perceive, and interpret their AF symptoms differently. Informed vigilance, from HCPs, could expedite the early detection of AF when women present with AF symptoms. Awareness of key differences by gender during this critical pre-diagnosis period can promote the development of strategies aimed specifically toward men and women in an effort to minimize the effects of a delayed AF diagnosis on QOL, morbidity, and mortality.
